# Growth factors regulate phospholipid biosynthesis in human fibroblast-like synoviocytes obtained from osteoarthritic knees

**DOI:** 10.1038/s41598-017-14004-9

**Published:** 2017-10-18

**Authors:** Katarzyna D. Sluzalska, Gerhard Liebisch, Jochen Wilhelm, Bernd Ishaque, Holger Hackstein, Gerd Schmitz, Markus Rickert, Juergen Steinmeyer

**Affiliations:** 1 Laboratory for Experimental Orthopaedics, Department of Orthopaedics, Justus Liebig University Giessen, Giessen, Germany; 2 Department of Clinical Chemistry and Laboratory Medicine, University Hospital Regensburg, Regensburg, Germany; 3 Medical Clinic II/IV, Justus-Liebig-University, Giessen, Germany; 4 Institute for Clinical Immunology and Transfusion Medicine, Justus Liebig University Giessen, Giessen, Germany

## Abstract

Elevated levels of growth factors and phospholipids (PLs) have been found in osteoarthritic synovial fluid (SF), although the metabolic regulation of PLs is currently unknown. This study aimed to determine the effects of growth factors on the biosynthesis of PLs by fibroblast-like synoviocytes (FLS) obtained from human osteoarthritic knee joints. Electrospray ionization tandem mass spectrometry was applied to analyse the newly synthesized PLs. In the presence of stable isotope-labelled PL precursors, cultured FLS were treated with either transforming growth factor-β1 (TGF-β1), bone morphogenetic protein (BMP)-2, BMP-4, BMP-7 or insulin-like growth factor-1 (IGF-1) alone or in combination with specific inhibitors of cell signalling pathways. TGF-β1 and IGF-1 markedly stimulated the biosynthesis of phosphatidylcholine (PC) before sphingomyelin (SM) and lysophosphatidylcholine (LPC) species were stimulated. BMPs elaborated less pronounced effects. The BMPs tested have different potentials to induce the biosynthesis of phosphatidylethanolamine (PE) and PE-based plasmalogens. Our study shows for the first time that TGF-β1 and IGF-1 substantially regulate the biosynthesis of PC, SM and LPC in human FLS. The functional consequences of elevated levels of PLs require additional study. The BMPs tested may be joint protective in that they upregulate PE-based plasmalogens that function as endogenous antioxidants against reactive oxygen species.

## Introduction

Osteoarthritis (OA) is a widespread degenerative joint disease which affects the entire articular joint including its cartilage, the subchondral bone and the synovium^1,2^. Synovial fluid (SF) analysis of OA knee joints has revealed altered levels, composition and a modified molecular weight distribution of the joint lubricants lubricin, hyaluronan and most phospholipid species compared to normal SF^3–5^. Nevertheless, the pathological mechanism responsible for the development and progression of OA is still a matter of huge scientific interest, and many studies have already investigated inter alia the role of pro-inflammatory cytokines such as IL-1β, TNFα, and growth factors during OA^6,7^.

The transforming growth factor β (TGF-β) superfamily has over 30 members including the TGF-β isoforms TGF-β1, -β2, -β3, the bone morphogenetic proteins (BMPs), the growth and differentiation factors (GDFs), the activins, the inhibins, and others^8^. The TGF-β superfamily signalling is initiated upon binding of a ligand to the type II receptor which later forms a complex with the type I receptor. The activated type I receptor can downstream the signal via non-canonical (PI3K) or canonical (SMAD) pathways^9^. The TGF-β subfamily TGF-β1, -β2, and -β3 isoforms activate receptor-regulated SMAD2 and 3, whereas the BMP subfamily members BMP-2, -4, and -7 activate SMAD1, 5 and 8. All three TGF-β isoforms bind exclusively to the type I receptor TGF-βR1, also known as ALK5. Other superfamily members can bind to two or more different types of receptors^9,10^.

It has been reported that the TGF-β superfamily members are intimately involved in the development of OA^9,11,12^. TGF-β isoforms are expressed in cartilage, bone and synovium, and tissue specific effects have also been observed. This growth factor participates in the regulation of chondrocyte maturation and hypertrophy during OA, promotes osteoblast maturation, and induces synovial tissue fibrosis^12,13^. Neutralization of excessive TGF-β with antibodies resulted in a decreased thickness of calcified cartilage, reduced proteoglycan loss and an attenuated degeneration of cartilage in a murine OA model^14^. Importantly, elevated TGF-β1 levels have been detected in synovial fluid taken from OA patients^15^.

Several studies have highlighted the contribution of BMPs to the pathogenesis of OA^16,17^. Although overexpression of BMP-2 in murine knee joints resulted in raised proteoglycan synthesis, it also induced degradation of aggrecan^18^. BMP-7 also stimulates the synthesis of aggrecan and collagen type II by chondrocytes but blocks the expression of matrix metalloproteinase-13, an enzyme which participates in cartilage destruction^19,20^. Elevated levels of BMP-2 were found in human OA as compared to normal cartilage^21^. Furthermore, the BMP-2 and BMP-7 levels in human plasma and synovial fluid were reported to correlate with OA severity.

The combination of BMP-7 with IGF-1 induced extracellular matrix production by normal and OA chondrocytes more markedly than either growth factor alone^22^. IGF-1 interacts with a specific receptor IGF-1R as well as with the insulin receptor. PI3K and ERK are the major signalling pathways via which IGF-1R regulates metabolism and gene expression^23,24^. Similar to other growth factors, IGF-1 induces anabolic effects and decreases catabolic responses in articular cartilage in addition to bestowing a protective effect on the synovium^11,25^. Also, IGF-1 has been found at markedly elevated levels in the serum and synovial fluid of human OA patients^26^.

In two recently published lipidomic studies^3,4^ we quantified 145 PLs and sphingolipids by quantitative mass spectrometric analysis of synovial fluid from patients with knee OA. We found that the concentrations of 97 lipids in early OA synovial fluid and 126 species in late OA SF were significantly higher than they were in control SF^3,4^. Lipids within SF are not only produced and secreted locally by e.g. FLS, but may also be derived in part from the circulation via blood vessels that are located mainly within the synovial capsule. Altered levels and relative compositions of certain PL species may alter the lubrication and inflammatory status of OA joints. Remarkably, the ether-based plasmalogens act as antioxidants which also stabilize and promote the hexagonal phase of PLs, thereby reducing the surface tension of PL mixtures^27^.

FLS are also known to synthesize other lubricants such as lubricin and hyaluronan^28,29^. The impacts of various growth factors on these other lubricants are known and may be of relevance for studying PL metabolism^5,30–36^. As one example, several studies have already investigated the effects of growth factors on lubricin using bovine articular cartilage and synovium^30–33^. TGF-β, IGF-1, and BMPs upregulated the accumulation of lubricin in chondrocytes and synoviocytes. Inhibition of TGF-β receptor type I with the specific inhibitor SB431242 abolished this effect. Synoviocytes were reported to be more sensitive to BMP subfamily members than articular chondrocytes. TGF-β was also found to stimulate the synthesis of hyaluronan in human synovial lining cells^34,35^.

Despite the functional role of growth factors in controlling the biosynthesis of the joint lubricants lubricin and hyaluronan by FLS, it can be assumed that growth factors are also involved in controlling PL production. However, the impact of growth factors on the synthesis of PLs by FLS is not currently known. The aim of our study was to investigate for the first time the individual roles which TGF-β1, IGF-1 and several BMPs have on PL classes and species synthesized by FLS obtained from human OA knee joints. Our findings provide insights into the mechanisms underlying the levels of local PL biosynthesis during OA. These mechanistic insights may identify new pharmacological targets for promoting lubrication and preventing OA progression.

## Materials and Methods

### Fibroblast-like Synoviocytes (FLS)

Human FLS were removed from synovial membranes of patients undergoing total knee replacement surgery as described elsewhere^29^. All procedures performed in the study involving human patients were in accordance with the principles outlined in the Declaration of Helsinki; approval by the local Ethical Review Committee of the Faculty of Medicine of the Justus Liebig University Giessen was obtained. All patients provided informed written consent to donate samples for research. The effects of recombinant growth factors on FLS were tested with cells derived from patients who fulfilled the following inclusion criteria: diagnosed OA (Kellgren-Lawrence scale 4), both genders (3 male, 2 female), age 50–85 years (80.2 ± 6.2 years), BMI 20–35 (28.6 ± 2.4 kg/m²), all CRP values (0.9 ± 0.4 mg/l). FLS were not obtained from patients with (a) other joint disease such as RA, gout, or trauma, (b) knee joint surgery within the 6 months prior to study onset, (c) severe diseases such as human immunodeficiency virus infection, tumour disease near to the joint, severe liver and kidney diseases, drug abuse and (d) intake of immunosuppressive drugs, corticosteroids or hyaluronan within the 6 months prior to study onset.

### Reagents

Unless otherwise indicated, all reagents were purchased from Sigma (Deisenhofen, Germany). Dulbecco’s modified Eagle media (DMEM), Dulbecco’s phosphate buffered saline (PBS) and penicillin/streptomycin were obtained from PAN Biotech (Aidenbach, Germany), and HPLC-grade methanol and chloroform were from Merck (Darmstadt, Germany). PL standards were purchased from Avanti Polar Lipids (Alabaster, AL, USA), and stable isotope labelled precursors of PLs from Cambridge Isotope Laboratories (Tewksbury, MA, USA).

### Cell Culture

FLS were cultured in a humidified 10% CO_2_ atmosphere at 37 °C using DMEM medium supplemented with 1.0 g/l glucose and 584 mg/l L-glutamine, 10% foetal bovine serum, 10 mM HEPES buffer, 10 U/ml penicillin and 0.1 mg/ml streptomycin. The experiments were performed with cells harvested at the end of passage 5. Routine test for mycoplasma contamination using the PCR Mycoplasma Test Kit I/C (PromoCell, Heidelberg, Germany) were negative.

### FACS Analysis

The purity of FLS was determined at the end of passage 5 using a BD FACSCANTO II flow cytometer (Becton Dickinson, Heidelberg, Germany). After trypsinization, cells were stained with APC anti-human CD90 (clone 5E10) and PE anti-human CD45 (clone 2D1) or APC mouse IgG1 (clone MOPC-21) and PE mouse IgG1 (clone MOPC-21) antibodies (BioLegend, San Diego, CA, USA). More than 90% of the cells used in the experiments stained positively for the fibroblast-specific antigen CD90 (96.4 ± 3.5%), whereas staining for CD45 was negative.

### Effect of Growth Factors on the Biosynthesis of PL

For analysis of PL biosynthesis, FLS of passage 5 were seeded into 6-well plates at a density of 80,000 cells per well. Cells were grown until 100% confluency and then starved for 24 hours in serine- and choline-depleted, phenol-free, DMEM medium (PAN Biotech, Aidenbach, Germany) containing 5% lipoprotein deficient serum (LPDS, gift of Dr. A. Sigruener), 10 mM HEPES buffer, 10 U/l penicillin, 0.1 mg/ml streptomycin, 4 mg/l folic acid and 42 mg/l L-serine. Afterwards, media were changed and cells were labelled with 225 µg/ml of [D9]-choline chloride and 25 µg/ml of [D4]-ethanolamine for 16 hours.

During this period, cells were simultaneously treated with 100 ng/ml BMP-2, BMP-4, or BMP-7, or 10 ng/ml TGF-β1, or 100 ng/ml IGF-1 (Peprotech, Rocky Hill, NJ, USA). The concentrations of growth factors and inhibitors tested were chosen based on data from literature^31–33^. Untreated FLS obtained from the same joints were used as controls. To investigate whether inhibition of TGF-βR1 kinase abolishes the effect of TGF-β1 on PL biosynthesis, cells were first pre-treated for 30 min with 10 µM of SB431542 (Selleckchem, Munich, Germany), and then stimulated with 10 ng/ml TGF-β1. To investigate the signal transduction mechanism of IGF-1, cells were first pre-treated for 30 min with 10 µM inhibitor of PI3Ks (LY294002; Selleckchem, Munich, Germany) or 1 µM inhibitor of ERK (SCH772984; Selleckchem, Munich, Germany), and then stimulated with 100 ng/ml IGF-1. Afterwards, cells were washed twice with 1x PBS and lysed with 0.2% sodium dodecyl sulphate (SDS). Wells were washed with distilled water and combined extracts were treated with ultrasound for 6 seconds at 40–50% power (Sonoplus, Bandelin Electronic GmbH, Berlin, Germany). The protein concentrations of cellular lysates were quantified using the Pierce™ BCA Protein Assay Kit (Thermo Fisher, Darmstadt, Germany).

Using identical culture conditions our preliminary experiments revealed that untreated FLS maintained a stable metabolism as indicated by their unaltered expression of the reference genes B2M, βactin, and GAPDH (QuantiTect^®^ Primer Assays, Qiagen, Hilden, Germany), their constant mitochondrial activity (Cell Titer 96^®^, Promega, Madison, WI, USA), and their high cell viability (>90%, trypan blue exclusion test, Sigma) and negligible caspase 3/7 activity (Caspase-Glo^®^ 3/7 Assay, Promega, Madison, WI, USA). The viability of FLS after 16 h of treatment with 10 ng/ml TGF-β or 100 ng/ml IGF-1 was 96.3 ± 3.3% respectively 92.3 ± 3.8% using trypan blue exclusion test. Also no change in cell morphology was observed after any treatment suggesting that FLS did not experience any cellular stress.

### Lipid Extraction and Mass Spectrometric Analysis

Lipid extraction was performed according to the procedure of Bligh and Dyer^37^ in the presence of non-naturally occurring internal lipid standards. Stable isotope-labelled and unlabelled PL species were quantified by electrospray ionization tandem mass spectrometry (ESI-MS/MS) on a Quattro Ultima™ Triple Quadruple mass spectrometer (Micromass, Wilmslow, UK) as described previously^38^. Briefly, a precursor ion scan of *m/z* 184 was used for phosphatidylcholine (PC), sphingomyelin (SM) and lysophosphatidylcholine (LPC) detection. [D9]-Choline-labelled lipids were analysed using a precursor ion scan of *m/z* 193. A neutral loss scan of 141 was used for phosphatidylethanolamine (PE) detection. [D4]-Ethanolamine-labelled lipids were analysed using a neutral loss scan of 145. Fragment ions of *m/z* 364, 390 and 392 were used for detecting phosphatidylethanolamine-based plasmalogens (PE P) PE P-16:0, PE P-18:1 and PE P-18:0. The isotopic overlap of lipid species was corrected and data analysis was performed using self-programmed Excel macros^39^. Lipid species were annotated according to a standard methodology for reporting lipid species identified by mass spectrometry^40^. Glycerophospholipid annotation is based on the assumption of even numbered carbon chains only. SM species annotation is based on the assumption that a sphingoid base with two hydroxyl groups is present. The quantitative values were normalized with respect to the cellular protein content and are expressed as nmol/mg or pmol/mg protein. Only PL species with concentrations higher than 1% of the corresponding PL class, and more than three times higher than the internal standard blank, were taken into account.

### Statistical Analysis of Data

**Figure 1:**
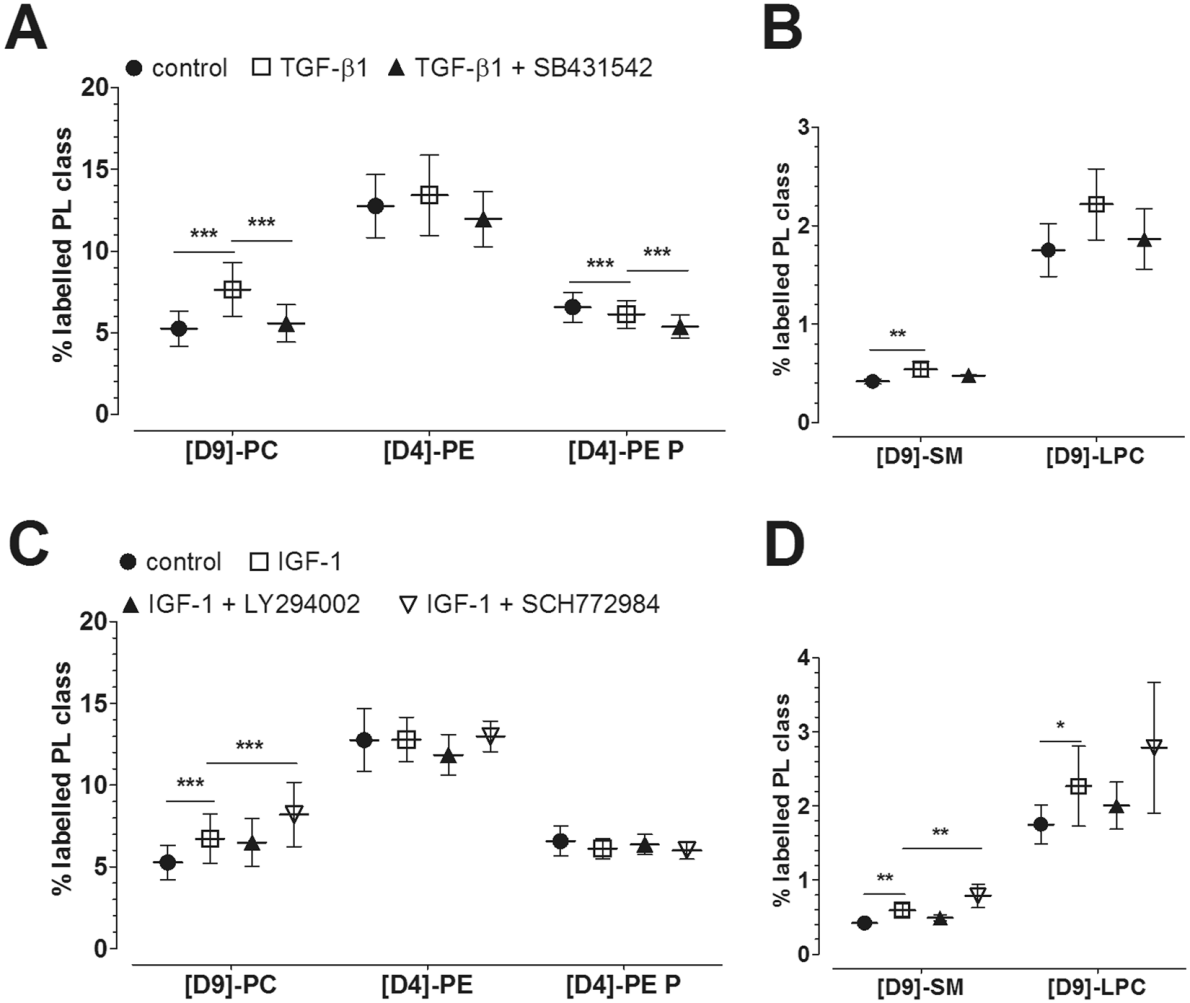
Effect of growth factors on the synthesis of PLs by human osteoarthritic FLS as modulated by inhibitors of cell signalling pathways. **(A**,**B)** Effect of TGF-β1 as modulated by TGF-βR1 inhibitor. The percentages of labelled PL classes from the total corresponding labelled and unlabelled PL classes are presented. FLS were first pre-treated for 30 min with 10 µM TGF-βR1 kinase inhibitor SB431542, and then treated with 10 ng/ml TGF-β1 for 16 hours in the presence of stable isotope-labelled PL precursors. (**C**,**D**) Effect of IGF-1 on PL biosynthesis as modulated by inhibitors of PI3K and ERK. The percentages of labelled PL classes from the total corresponding labelled and unlabelled PL classes are presented. FLS were first pre-treated for 30 min with 10 µM LY294002 inhibitor (PI3K) or 1 µM SCH772984 inhibitor (ERK), and then treated with 100 ng/ml IGF-1 for 16 hours in the presence of stable isotope-labelled PL precursors. Data are expressed as means ± SDs (n = 5). *p ≤ 0.05; **p ≤ 0.01; ***p ≤ 0.001. PC = phosphatidylcholines; PE = phosphatidylethanolamines; PE P = phosphatidylethanolamine-based plasmalogens; SM = sphingomyelins; LPC = lysophosphatidylcholines.

**Figure 2:**
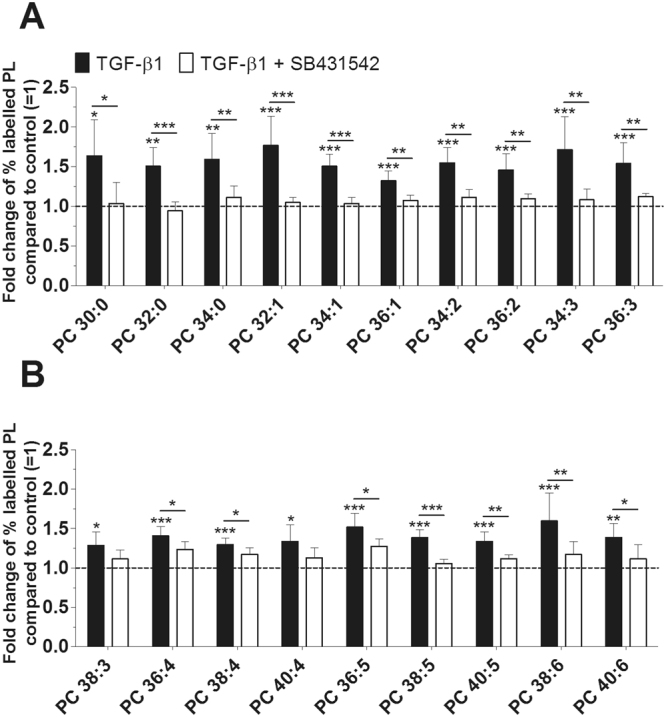
Effect of TGF-β1 on the biosynthesis of PC species as modulated by TGF-βR1 inhibitor. PC synthesis was monitored by ESI-MS/MS in the presence of 10 ng/ml TGF-β1 (black bars) with or without 10 µM TGF-βR1 inhibitor SB431542 (white bars) for 16 hours (n = 5). The percentages of labelled PL species were calculated and then normalized as a ratio compared to the corresponding untreated control. Data are expressed as means ± SDs of the x-fold change of % labelled PL species compared to untreated controls (=1). *p ≤ 0.05; **p ≤ 0.01; ***p ≤ 0.001; TGF-β1 versus control, or TGF-β1 + SB431542 versus TGF-β1. PC = phosphatidylcholine.

**Figure 3:**
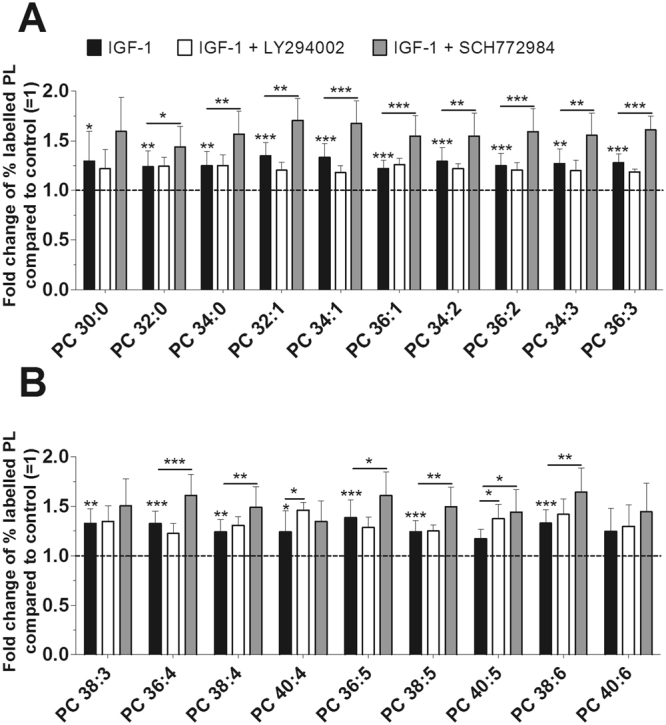
Effect of IGF-1 on the biosynthesis of PC species as modulated by inhibitors of PI3K and ERK. PL synthesis was quantified by mass spectrometry in the presence of 100 ng/ml IGF-1 (black bars) with or without 10 µM LY294002 inhibitor (PI3K, white bars) or 1 µM SCH772984 inhibitor (ERK, grey bars) for 16 hours (n = 5). The percentages of labelled PC species were calculated and then normalized as a ratio of the corresponding untreated control. Data are expressed as means ± SDs of the x-fold change of % labelled PC species compared to untreated controls (=1). *p ≤ 0.05; **p ≤ 0.01; ***p ≤ 0.001; IGF-1 versus control, or IGF-1 + LY294002 versus IGF-1, or IGF-1 + SCH772984 versus IGF-1. PC = phosphatidylcholine.

**Figure 4:**
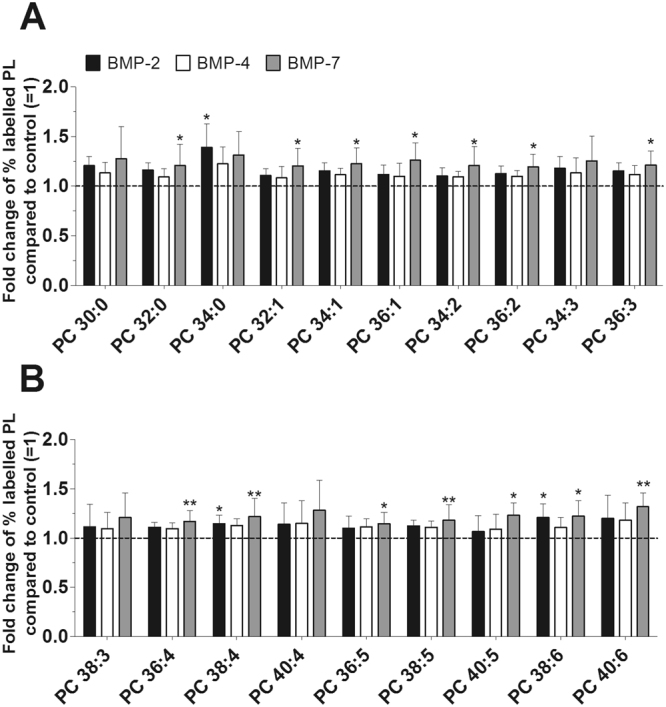
Effect of BMPs on the biosynthesis of PC species. PC synthesis was determined with ESI-MS/MS in the presence of 100 ng/ml BMP-2 (black bars), 100 ng/ml BMP-4 (white bars) or 100 ng/ml BMP-7 (grey bars) for 16 hours (n = 5). The percentages of labelled PL species were calculated and then normalized as a ratio of the corresponding untreated control. Data are expressed as means ± SDs of the x-fold change of % labelled PL species compared to untreated controls ( =1). *p ≤ 0.05; **p ≤ 0.01; BMP-2, or BMP-4, or BMP-7 versus control. PC = phosphatidylcholine.

Each experimental condition was repeated 4 times using FLS obtained from 5 patients (N = 5). The %-values quoted in the text within brackets represent the percentage of labelled PL class or species from the total corresponding PL class or species being determined as labelled and unlabelled PL. The data were analyzed as logits of the proportions in a two-factorial linear model. The factor “Patient” accounts for systematic differences between cell cultures obtained from different patients (“paired analysis”). The “Group” factor accounts for differences between treatments. Residual diagnostic plots showed good agreement of the data with the model assumptions. Differences in treatment effects were tested with Tukey’s HSD (Figs 1–4, Tables 1 and 2). The analysis was performed in R 3.3.2^41^. Graphics were created using Prism 5.2 (GraphPad Software Inc., La Jolla, CA, USA). Data are presented as means and standard deviations. Stars indicate the significance of comparisons (*p < 0.05; **p < 0.01; ***p < 0.001).

**Table 1:** Effect of BMPs on the biosynthesis of PL classes.

Labelled PL class	Control [%]	BMP-2 [%]	BMP-4 [%]	BMP-7 [%]
[D9]-PC	4.45 ± 0.93	5.03 ± 1.02	4.92 ± 1.17	5.29 ± 0.94**
[D4]-PE	9.83 ± 1.88	11.0 ± 2.48	10.6 ± 1.80	11.5 ± 1.93*
[D4]-PE P	5.25 ± 0.53	5.78 ± 0.89**	5.44 ± 0.54	5.78 ± 0.56**
[D9]-SM	0.30 ± 0.02	0.34 ± 0.03	0.33 ± 0.03	0.36 ± 0.06
[D9]-LPC	1.65 ± 0.20	1.56 ± 0.18	1.87 ± 0.48	1.78 ± 0.26

FLS were treated with 100 ng/ml BMP-2, BMP-4 or BMP-7 for 16 hours in the presence of the stable isotope-labelled precursors of PLs, namely [D9]-choline and [D4]-ethanolamine. The percentages of labelled PL class from the total corresponding labelled and unlabelled PL class are presented. Data are presented as means ± SDs (n = 5). *p ≤ 0.05, BMP-2, or BMP-4, or BMP-7 versus control; **p ≤ 0.01, BMP-2, or BMP-4, or BMP-7 versus control. PC = phosphatidylcholines; PE = phosphatidylethanolamines; PE P = phosphatidylethanolamine-based plasmalogens; SM = sphingomyelins; LPC = lysophosphatidylcholines.

**Table 2:** Effect of BMPs on the newly synthesized PE and PE P species.

PL specie	Fold change of % labelled PL compared to control ( = 1)		
	BMP-2	BMP-4	BMP-7
PE 36:1	1.22 ± 0.15*	1.15 ± 0.13	1.28 ± 0.20**
PE 36:2	1.16 ± 0.15	1.09 ± 0.10	1.27 ± 0.19*
PE 38:3	1.12 ± 0.14	1.06 ± 0.13	1.24 ± 0.19*
PE 40:5	1.18 ± 0.13*	1.07 ± 0.12	1.18 ± 0.17*
PE 40:6	1.19 ± 0.11**	1.15 ± 0.17*	1.27 ± 0.17***
PE P-18:0/18:1	1.19 ± 0.11*	1.27 ± 0.14**	1.13 ± 0.12
PE P-16:0/20:4	1.19 ± 0.09**	1.08 ± 0.06	1.19 ± 0.07**
PE P-18:0/20:4	1.15 ± 0.10*	1.07 ± 0.10	1.15 ± 0.09*
PE P-18:1/20:4	1.08 ± 0.07*	1.01 ± 0.06	1.09 ± 0.06*

FLS were treated with 100 ng/ml BMP-2, BMP-4 or BMP-7 for 16 hours in the presence of the stable isotope-labelled precursors of PLs, namely [D9]-choline and [D4]-ethanolamine. The table presents only species which were affected by BMPs. The percentages of labelled PL species from the total corresponding labelled and unlabelled PL class were calculated before being normalized as a ratio to the corresponding untreated controls (=1). Data are presented as means ± SDs (n = 5). *p ≤ 0.05, BMP-2, or BMP-4, or BMP-7 versus control; **p ≤ 0.01, BMP-2, or BMP-4, or BMP-7 versus control; ***p ≤ 0.001, BMP-2, or BMP-4, or BMP-7 versus control. PE = phosphatidylethanolamine; PE P = phosphatidylethanolamine-based plasmalogen.

### Data Availability

The datasets generated during the current study are available from the corresponding author on reasonable request.

## Results

### Effect of TGF-β1 on the Biosynthesis of PL Classes

Mass spectrometry analysis revealed that TGF-β1 increased the synthesis of PC (7.65 ± 1.63%, p ≤ 0.001; 5.86 ± 1.16 nmol/mg protein) to 146% when compared to untreated controls (5.25 ± 1.07%; 4.31 ± 0.84 nmol/mg protein) (Fig. 1A; Suppl. Table 1). SM synthesis (0.54 ± 0.08%, p = 0.009; 130 ± 20 pmol/mg protein) was also elevated to 129% when compared to untreated controls (0.42 ± 0.02%; 110 ± 20 pmol/mg protein). Moreover, the synthesis of LPC (2.22 ± 0.36%, p = 0.068; 22 ± 4 pmol/mg protein) was slightly increased to 127% when compared to untreated control (1.75 ± 0.27%, p = 0.068; 20 ± 7 pmol/mg protein) cultures (Fig. 1B; Suppl. Table 1). Since SM and LPC are derived from PC, the ratio of newly synthesized SM or LPC to newly synthesized PC was calculated. Analysis revealed comparable SM/PC and LPC/PC ratios in treated versus untreated FLS indicating that elevated SM and LPC levels were derived from the increased synthesis of its precursor PC. Inhibition of TGF-βR1 with SB431542 abolished the stimulatory TGF-β1 effect on PC biosynthesis (p ≤ 0.001). Addition of SB431542 inhibitor slightly decreased the biosynthesis of PE-based plasmalogen to 88% when compared to sole TGF-β1 treatment (TGF-β1 versus control, p = 0.001; TGF-β1 + SB431542 versus TGF-β1, p ≤ 0.001; Fig. 1A; Suppl. Table 1). PE biosynthesis remained unaffected. 86% of newly synthesized PC species were mostly unsaturated in untreated and TGF-β1 treated FLS. The lengths of the FA chains of newly synthesized PC species were similar in treated and untreated FLS and were as follows: 77.2 ± 4.9% had ≤ 36 carbon atoms in the untreated controls, while 80.4 ± 4.6% had ≤ 36 carbon atoms after treatment with TGF-β1.

### Effect of IGF-1 on the Biosynthesis of PL Classes

As shown in Fig. 1C and D, synthesis of PL was also stimulated by IGF-1. This growth factor increased the synthesis of PC to 128% (6.71 ± 1.53%, p ≤ 0.001; 5.06 ± 1.06 nmol/mg protein) compared to untreated controls (5.25 ± 1.07%; 4.31 ± 0.84 nmol/mg protein), of SM to 141% (0.59 ± 0.09%, p = 0.002; 140 ± 30 pmol/mg protein) versus controls (0.42 ± 0.02%; 110 ± 20 pmol/mg protein), and of LPC to 130% (2.27 ± 0.54%, p = 0.031; 22 ± 4 pmol/mg protein) compared to untreated controls (1.75 ± 0.27%; 20 ± 7 pmol/mg protein) (Suppl. Table 1). However, the ratio of newly synthesized SM or LPC to newly synthesized PC did not change, indicating that the observed effects on SM and LPC were not specific for IGF-1. Unexpectedly, inhibition of ERK with the ERK inhibitor SCH772984 in the presence of IGF-1 more strongly induced the synthesis of PC to 156%, and subsequently of SM to 188% and of LPC to 159% when compared to untreated controls (Fig. 1C,D, Suppl. Table 1). Again, calculation of the ratio of SM or LPC to PC revealed that the IGF-1 effect on SM and LPC was indirect in that they resulted from the elevated synthesis of the precursor PC. In addition, IGF-1 displayed no marked effect on PE and PE-based plasmalogen synthesis. Again, 86% of newly synthesized PC species were unsaturated in the untreated or IGF-1 treated FLS. The lengths of the FA chains of newly synthesized PC species were similar in treated and untreated FLS, i.e. 77.2 ± 4.9% had ≤ 36 carbon atoms in the untreated controls and 79.6 ± 3.6% had ≤ 36 carbon atoms after treatment with IGF-1.

### Effect of BMPs on PL Biosynthesis

Since certain members of the BMP subfamily were reported to be involved in OA progression and lubricin FLS synthesis, we screened BMP-2, -4 and -7 for their effects on PL production by human OA FLS. As shown in Table 1, the effects of BMPs on PL biosynthesis were rather weak, but nevertheless specific. BMP-2 increased the biosynthesis of PE P to 110% (5.78 ± 0:89%, p = 0.008; 2.36 ± 0.41 nmol/mg protein) when compared to untreated controls (5.25 ± 0.53%; 2.28 ± 0.46 nmol/mg protein; Suppl. Table 1). Furthermore, BMP-7 stimulated the biosynthesis of PC to 118% (5.29 ± 0.94%, p = 0.010; 4.22 ± 0.77 nmol/mg protein), of PE to 117% (11.5 ± 1.93%, p = 0.012; 2.38 ± 0.58 nmol/mg protein), and of PE-based plasmalogen to 110% (5.78 ± 0.56%, p = 0.006; 2.47 ± 0.39 nmol/mg protein) versus untreated controls (4.45 ± 0.93%; 3.50 ± 0.72 nmol/mg protein, 9.83 ± 1.88%; 2.07 ± 0.47 nmol/mg protein, and 5.25 ± 0.53%; 2.28 ± 0.46 nmol/mg protein, respectively; Suppl. Table 1). However, BMP-4 did not display any marked effect on the synthesis of any specific PL class. Also, 87.0 ± 0.5% of newly synthesized PC species were unsaturated irrespective of treatment with BMPs. The lengths of the FA chains of newly synthesized PC species were similar in untreated versus BMP treated FLS and were as follows: 76.0 ± 5.4% had ≤ 36 carbon atoms in the untreated controls and 75.8 ± 5.2%, 76.2 ± 5.1%, 76.0 ± 4.7% had ≤ 36 carbon atoms after treatment with BMP-2, BMP-4, and BMP-7, respectively.

### Detailed Analysis of the TGF-β1 Effect on PL Species

Our further studies focused on whether TGF-β1 specifically affects the biosynthesis of PL species, and whether the known TGF-β signal transduction pathway is involved in PL synthesis. Inhibition of TGF-βR1 kinase ALK 5 with SB431542 abolished the effect of TGF-β1 on the synthesis of 17 PC species (Fig. 2A,B). The concentrations of newly synthesized PC species varied between 35 ± 12 pmol/mg protein (PC 40:4) and 812 ± 142 pmol/mg protein (PC 34:1) for untreated controls and between 39 ± 12 pmol/mg protein (PC 40:4) and 1189 ± 205 pmol/mg protein (PC 34:1) after treatment with TGF-β1 (Suppl. Table 2). As expected, the most abundant PC species was PC 34:1. TGF-β1 significantly enhanced the biosynthesis of all 19 PC species by between 130% and 180% when compared to untreated controls (p-values from 0.023 to <0.001).

Our ESI-MS/MS analysis also allowed us to characterize ten newly synthesized SM species. The concentrations of SM varied between 3 ± 1 pmol/mg protein (SM 36:2) and 54 ± 10 pmol/mg protein (SM 34:1) for untreated controls and between 4 ± 1 pmol/mg protein (SM 36:2) and 69 ± 17 pmol/mg protein (SM 34:1) where treatment was carried out with TGF-β1 (Suppl. Table 2).

However, the biosynthesis of individual PL species containing ethanolamine was barely affected by TGF-β1. Only PE 36:3 was markedly elevated to 124% when compared to untreated controls (p = 0.018). TGF-β1 had no or negligible effects on the synthesis of PE-based plasmalogen species (data not shown).

### Detailed Analysis of PL Species as Modulated by IGF-1

Our in-depth analysis aimed to investigate whether certain PL species are individually affected by IGF-1. The concentrations of newly synthesized PC species varied between 35 ± 12 pmol/mg protein (PC 40:4) and 812 ± 142 pmol/mg protein (PC 34:1) for untreated controls, and between 33 ± 9 pmol/mg protein (PC 40:4) and 1036 ± 120 pmol/mg protein (PC 34:1) after treatment with IGF-1 (Suppl. Table 2). Out of the 19 PC species that were detected, IGF-1 significantly enhanced the synthesis of 17 PC species from between 120% and 140% when compared to untreated controls (p-values from 0.039 to <0.001). Unexpectedly, inhibition of PI3K with LY294002 stimulated the synthesis of PC 40:4 to 146% and PC 40:5 to 138%. Also, blockade of ERK with the specific inhibitor SCH772984 even enhanced the synthesis of 15 PC species from between 144% (PC 40:5) and 170% (PC 32:1) compared to untreated controls (Fig. 3A,B).

Furthermore, the concentrations of newly synthesized SM species varied between 3 ± 1 pmol/mg protein (SM 36:2) and 54 ± 10 pmol/mg protein (SM 34:1) for untreated controls, and between 3 ± 1 pmol/mg protein (SM 36:2) and 70 ± 17 pmol/mg protein (SM 34:1) after exposure to IGF-1 (Suppl. Table 2). In addition, IGF-1 had no or only negligible effects on the synthesis of PE and PE-based plasmalogen species (data not shown).

### Detailed Analysis of the BMPs Effects on PL Species

Since our cultured FLS only weakly responded to BMPs, we performed an in-depth analysis of the PL species composition. The concentrations of newly synthesized PC species varied between 28 ± 6 pmol/mg protein (PC 34:3) and 649 ± 130 pmol/mg protein (PC 34:1) for untreated controls and between 32 pmol/mg protein (PC 40:4) and 797 pmol/mg protein (PC 34:1) after treatment with BMPs (Suppl. Table 3). BMP-2 significantly enhanced the synthesis of 3 PC species: to 139% for PC 34:0 (p = 0.029), to 114% for PC 38:4 (p = 0.035), and to 121% for PC 38:6 (p = 0.045), while BMP-4 had no effect on the biosynthesis of any PC species. BMP-7 enhanced a total of 14 PC species from between 115% (PC 36:5, p = 0.026) and 132% (PC 40:6, p = 0.004) compared to untreated controls (Fig. 4A,B).

Mass spectrometric analysis of newly synthesized SM species revealed that they only existed at low levels, similar to those seen after treatment with TGF-β1 and IGF-1 (Suppl. Tables 2 and 3).

The three investigated BMPs differentially regulated the biosynthesis of 13 PE and 19 PE-based plasmalogen species. Table 2 shows only those species which were markedly regulated by any of the three tested BMPs. Two PE species were exclusively enhanced by BMP-7 (PE 36:2, p = 0.010 and PE 38:3, p = 0.018). BMP-2 and BMP-7 enhanced PE 36:1 (p = 0.011 and p = 0.003, respectively), PE 40:5 (p = 0.013 and p = 0.018, respectively), PE P-16:0/20:4 (p = 0.001 and p = 0.001, respectively), PE P-18:0/20:4 (p = 0.016 and p = 0.018, respectively), and PE P-18:1/20:4 (p = 0.024 and p = 0.012, respectively), whereas all three BMPs increased the synthesis of PE 40:6 (p = 0.006, p = 0.035, and p = 0.001, respectively). The synthesis of PE P-18:0/18:1 was increased by BMP-2 (p = 0.030) and BMP-4 (p = 0.004) (Table 2).

## Discussion

Growth factors have been reported to regulate the synthesis of the joint lubricants hyaluronan and lubricin in FLS^30–32,34,35^, but the impact of these factors on the production of PLs that are also involved in joint lubrication is unknown. The objective of this study was to determine the effects of TGF-β1, IGF-1 and BMP-2, -4 and -7 on the synthesis of PLs by human FLS obtained from OA knee joints. These growth factors were chosen since they were reported to be present at elevated levels in OA SF^15,26,42–44^. Using sophisticated ESI-MS/MS analysis, our study demonstrates the critical role growth factors play in the regulation of PL production by human OA FLS. Our previous studies using a large OA cohort already revealed that the level of lipid species in SF is not dependent on age or gender^3,4^. Thus it appears unlikely that the biosynthesis of lipids is confounded by age or gender of the patients.

The five growth factors investigated differ markedly with respect to their impact on PC synthesis by human FLS, supporting the concept of a sophisticatedly regulated PL biosynthesis. TGF-β1 most strongly stimulated the PC synthesis followed by IGF-1, whereas BMP-2 and -7 elaborated less intense effects. We used 100% confluent FLS to ensure that elevated lipid synthesis could be attributed solely to the impact of growth factors rather than cell division. We chose a labelling period of 16 hours to determine the biosynthesis of PLs. A longer labelling period would have allowed us to observe larger differences. However, simultaneously newly synthesized PLs may also be slowly degraded and released or recycled. Thus, a short-term labelling period was chosen to determine the biosynthesis of PLs solely as being modulated by growth factors. Our data suggest that TGF-β1 and IGF-1 in particular are important regulators also of polyunsaturated PC species which are PLs that are also known to be involved in joint lubrication^45^.

Our investigation may have clinical implications in that similar PC species that are altered in early and late OA SF^3^ are also synthesized at elevated levels in FLS treated with growth factors. 19 PC species were enhanced during early OA, and 18 of these were increased even further during late OA. Remarkably, the biosynthesis of 16 and 13 PC species found to be elevated in OA SF were also stimulated in FLS by TGF-β1 and IGF1, respectively. In addition, 3 and 12 PC species were enhanced in OA SF as well as after treatment with BMP-2 and BMP-7, respectively. Interestingly, four PC species (PC 34:1, PC 36:2, PC 36:3 and PC 36:4) that were upregulated by TGF-β1, IGF-1, and BMP-7 were also found to be elevated in SF during early and late OA. In summary, our comparison implies that growth factors, present at elevated levels in OA SF^30–32,34,35^, can induce FLS to produce more PC, some species of which are also enhanced in human OA SF.

PC species are the most abundant PLs in eukaryotic cell membranes, but whether FLS also preferentially contribute polyunsaturated PC species to SF so that joint lubrication can be improved requires additional study. Also, the majority of PC species and metabolites (e.g. phosphatidic acid and diacylglycerol) produced by FLS can be used as precursors for other lipids, or may participate in cell signalling^46–48^. However, the metabolic consequences of the elevated levels and biosynthesis of PC species quantified during OA and after growth factor exposure remain to be elucidated.

Our study shows that elevated levels of SM and LPC species were determined in FLS after treatment with TGF-β1 and IGF-1. The effects of these growth factors on these two PL classes were not specific since both factors also elevated PC which is the biosynthetic precursor for SM. As such, PC will have some impact on many metabolic pathways that constitute the SM cycle. Like PC, SM serves as a building block for cell membranes and is a major source of ceramides and its metabolite sphingosine-1-phosphate (S1P) which arises due to the action of sphingomyelinases. Some of these metabolites, including ceramides and S1P, also play important roles in cell signalling, cell differentiation, survival, and apoptosis^49–52^.

PC is also the precursor of LPC and phosphatidic acid, all of which have been reported as having substantial signalling functions. As one example, LPC have been found to be important molecules in cell signalling pathways such as MAPK/ERK, PI3K and Rho, and have some pro-inflammatory properties including the activation of macrophages, a process which involves the elevated expression of IL-1ß, urokinase-type plasminogen activator and its receptor^53–55^. Taken together, these data indicate multiple functions induced by elevated biosynthesis and levels of SM and LPC species in OA FLS and SF, but their precise biological role during OA is still not known.

BMPs demonstrated agent-specific stimulatory effects on some PE and PE-based plasmalogen species, suggesting that there is a division in tasks among various members of the BMP subfamily with effect sizes ranging from weak to quite strong. Together with other lipids in the membrane bilayer, PE is believed to stabilize membrane proteins in their optimum conformation and to ensure a correct tertiary structure so that membranes involved in transport can function correctly^56,57^. Some PE species have been reported to play pivotal roles during cell division, or to be precursors of other bioactive molecules^46,58,59^. Further studies will certainly provide enlightenment as to their definitive role during OA.

All three BMPs elevated the levels of PE-based plasmalogen species that are known to be protective against the devastating effects of reactive oxygen species on cell membrane PLs^60,61^. Remarkably, elevated levels of PE-based plasmalogens were also found in OA synovial fluid, an observation supporting the concept of a protective function of these PLs during OA^3^. However, TGF-β1 and IGF-1 had no or just negligible effects on these oxygen scavenging plasmalogens.

Our experiment using the specific TGF-βR1 kinase inhibitor SB431542 demonstrates TGF-β receptor-1 signalling as a key factor in PC biosynthesis. Blocking of endogenous TGF signalling abolished the enhanced biosynthesis of 17 of 19 PC species, even though other pathways such as the non-canonical signalling pathway PI3K are known to exist. Whether PI3K or ERK are involved in IGF-1-induced PL biosynthesis remains to be determined since their inhibition actually enhanced the biosynthesis of 2 or 17 PC species, respectively. We cannot exclude an inhibitor specific effect. Also, IGF-1 may act on PLs through other non-canonical signalling pathways, although this remains to be confirmed.

Interestingly, TGF-β1 acts differently on the pulmonary production of surfactant which is a surface-active mixture of PLs and proteins secreted by alveolar type II epithelial cells^62,63^. The surface tension at the air liquid interface is reduced by the surfactant, thus ensuring alveolar stability at low lung volumes. The predominant PL synthesized there is dipalmitoylphosphatidylcholine (DPPC, PC 32:0), which is the major contributor to the surface activity^62^. In SF this PL is also present, but the majority of PC species in SF have 34 or more C-atoms that are unsaturated e.g. PC 34:1, PC 34:2, PC 36:1, PC 36:2, and PC 36:3: these may be the major PLs contributing to joint lubrication^3^. Beers *et al*. reported that TGF-β1 inhibits surfactant proteins, fatty acid synthetase expression and [^3^H]-choline incorporation into PC species of human foetal lung explants^63^. Moreover, epithelial cells treated with TGF-β1 did not develop the lamellar bodies that represent the vehicles of lipid secretion. Taken together, these data indicate that the surfactant system of the lung is regulated differently than is the lubricating system present within articular joints.

In conclusion, our study demonstrates that TGF-β1 and IGF-1 are growth factors with major stimulatory impacts on PC synthesis within human OA FLS. Elevated PC species were found in human OA SF, and are the biosynthetic precursors for the increased levels of SM and LPC species that are reported to be involved in mediating various intra- and intercellular effects in other cells. Depending on the BMP tested, elevated levels of various PE-based plasmalogens were measured, indicating that BMPs have different potentials to act protectively against the destructive effects of reactive oxygen species. Our study demonstrates TGF-β-receptor-1 signalling as the major pathway via which TGF-β1 regulates PC biosynthesis. Comparison with the effects of TGF-β1 on the pulmonary production of surfactant reveals that articular joints use other cellular mechanisms to control their lubricating system. However, the exact biological functions of elevated levels of PL species during OA remain to be elucidated. This study was designed to contribute a considerable amount of new knowledge about the regulation of PL metabolism in articular joints, and may further support the quest to identify new targets for pharmacological agents.

## Electronic supplementary material

Supplementary Tables 1–3
