# Supplementary material for: Growth factors regulate phospholipid biosynthesis in human fibroblast-like synoviocytes obtained from osteoarthritic knees
Source: Sci Rep. 2017 Oct 18;7:13469. doi: 10.1038/s41598-017-14004-9 (PMC5647370; doi:10.1038/s41598-017-14004-9)
Supplement: Supplementary file 1 — Supplementary Tables 1–3 [file 41598_2017_14004_MOESM1_ESM.pdf]

# Supplementary Informations

## Growth factors regulate phospholipid biosynthesis in human fibroblast-like synoviocytes obtained from osteoarthritic knees

Katarzyna D. Sluzalska[1], Gerhard Liebisch[2], Jochen Wilhelm[3], Bernd Ishaque[1], Holger Hackstein[4], Gerd Schmitz[2], Markus Rickert[1], & Juergen Steinmeyer[1*]

[1]Katarzyna D. Sluzalska, MSc, Bernd Ishaque, MD, Markus Rickert, MD, and Juergen Steinmeyer, PhD: Laboratory for Experimental Orthopaedics, Department of Orthopaedics, Justus Liebig University Giessen, Germany

[2]Gerhard Liebisch, PhD, Gerd Schmitz, MD: Department of Clinical Chemistry and Laboratory Medicine, University Hospital Regensburg, Germany

[3]Jochen Wilhelm, PhD: Medical Clinic II/IV, Justus-Liebig-University, Giessen, Germany

[4]Holger Hackstein, MD: Institute for Clinical Immunology and Transfusion Medicine, Justus Liebig University Giessen, Germany

| **Treatment** | **[D9]-PC** [nmol/mg] | **[D4]-PE** [nmol/mg] | **[D4]-PE P** [nmol/mg] | **[D9]-SM** [pmol/mg] | **[D9]-LPC** [pmol/mg] |
|---|---|---|---|---|---|
| **control** | 4.31±0.84 | 2.74±0.50 | 2.78±0.53 | 110±20 | 20±7 |
| TGF-β1 | **5.86±1.16** | 3.02±0.55 | **2.47±0.50** | **130±20** | 22±4 |
| TGF-β1 + SB431542 | **4.19±0.82** | 2.56±0.46 | **2.15±0.34** | 120±20 | 22±4 |
| IGF-1 | **5.06±1.06** | 2.84±0.50 | 2.39±0.34 | **140±30** | **22±4** |
| IGF-1 + LY294002 | 5.28±1.25 | 2.63±0.45 | 2.68±0.31 | 120±20 | 24±5 |
| IGF-1 + SCH772984 | **6.07±1.51** | 3.01±0.54 | 2.35±0.34 | **190±60** | 30±7 |
| **control** | 3.50±0.72 | 2.07±0.47 | 2.28±0.46 | 80±10 | 20±0 |
| BMP-2 | 3.85±0.72 | 2.25±0.69 | **2.36±0.41** | 80±10 | 20±0 |
| BMP-4 | 3.81±0.97 | 2.21±0.51 | 2.29±0.32 | 80±20 | 24±5 |
| BMP-7 | **4.22±0.77** | **2.38±0.58** | **2.47±0.39** | 90±10 | 22±4 |

**Supplementary Table 1: Effect of growth factors on the level of newly synthesized PL classes.** The quantitative values obtained from stable isotope-labelled PL classes were normalized with respect to the cellular protein content and expressed as nmol/mg protein or pmol/mg protein. Data are presented as means ± SDs (n=5). Bold data correspond to the significantly altered changes being expressed as %-labelled PL as shown in Figure 1 and Table 1. PC = phosphatidylcholine; PE = phosphatidylethanolamine; PE P = phosphatidylethanolamine-based plasmalogens; SM = sphingomyelin; LPC = lysophosphatidylcholine.

| PL specie | control | | TGF-β1 | | IGF-1 | |
|---|---|---|---|---|---|---|
| | [pmol/mg] | [% labelled PL] | [pmol/mg] | [% labelled PL] | [pmol/mg] | [% labelled PL] |
| PC 30:0 | 46±16 | 4.3±1.4 | 73±21 | **6.6±1.6** | 56±13 | **5.3±1.3** |
| PC 32:0 | 426±105 | 5.7±1.2 | 584±200 | **8.6±2.3** | 477±137 | **7.0±1.3** |
| PC 34:0 | 59±22 | 5.4±1.5 | 82±36 | **8.4±2.4** | 65±27 | **6.6±1.5** |
| PC 32:1 | 148±27 | 5.1±0.9 | 267±60 | **8.8±2.3** | 193±24 | **6.8±1.5** |
| PC 34:1 | 812±142 | 5.3±1.3 | 1189±205 | **8.0±2.3** | 1036±120 | **7.1±2.0** |
| PC 36:1 | 208±33 | 3.3±0.7 | 255±42 | **4.3±0.9** | 230±26 | **4.0±1.0** |
| PC 34:2 | 332±58 | 8.0±1.6 | 527±101 | **12.4±2.8** | 419±67 | **10.3±2.3** |
| PC 36:2 | 315±27 | 5.2±1.4 | 438±64 | **7.5±2.1** | 379±52 | **6.4±1.7** |
| PC 34:3 | 37±6 | 10.0±2.3 | 74±25 | **16.6±3.9** | 46±7 | **12.5±2.4** |
| PC 36:3 | 210±37 | 7.0±1.8 | 309±67 | **10.6±2.6** | 254±72 | **9.1±2.8** |
| PC 38:3 | 78±25 | 4.3±1.0 | 88±24 | **5.4±1.1** | 87±22 | **5.6±1.2** |
| PC 36:4 | 350±122 | 5.9±0.7 | 435±145 | **8.3±0.9** | 428±167 | **7.8±1.3** |
| PC 38:4 | 448±182 | 4.6±0.8 | 506±194 | **5.9±0.8** | 479±190 | **5.7±0.9** |
| PC 40:4 | 35±12 | 6.3±1.6 | 39±12 | **8.3±2.0** | 33±9 | **7.6±1.6** |
| PC 36:5 | 43±12 | 6.5±1.1 | 60±15 | **9.8±1.2** | 51±16 | **8.9±1.3** |
| PC 38:5 | 282±93 | 6.5±1.2 | 337±105 | **9.0±1.3** | 302±105 | **8.1±1.5** |
| PC 40:5 | 54±15 | 5.7±1.2 | 62±17 | **7.6±1.4** | 51±15 | 6.7±1.5 |
| PC 38:6 | 65±18 | 5.5±1.1 | 83±19 | **8.5±1.2** | 68±24 | **7.3±1.6** |
| PC 40:6 | 42±15 | 5.6±1.4 | 49±12 | **7.6±1.4** | 42±13 | 6.8±1.4 |
| SM 32:1 | 6±1 | 1.3±0.2 | 6±0 | 1.5±0.2 | 6±1 | 1.5±0.3 |
| SM 33:1 | 5±2 | 0.8±0.2 | 5±1 | 0.9±0.2 | 4±1 | 0.8±0.1 |
| SM 34:0 | 5±2 | 0.5±0.2 | 6±2 | 0.7±0.2 | 7±3 | 0.8±0.3 |
| SM 34:1 | 54±10 | 0.4±0.0 | 69±17 | **0.5±0.1** | 70±17 | **0.5±0.1** |
| SM 34:2 | 5±1 | 1.4±0.2 | 5±1 | 1.5±0.3 | 5±1 | 1.6±0.4 |
| SM 35:2 | 6±1 | 0.8±0.1 | 7±1 | 1.0±0.2 | 9±2 | **1.2±0.1** |
| SM 36:1 | 4±2 | 0.3±0.1 | 6±1 | 0.4±0.1 | 7±2 | 0.5±0.1 |
| SM 36:2 | 3±1 | 1.8±0.3 | 4±1 | **2.4±0.4** | 3±1 | 2.0±0.3 |
| SM 42:1 | 8±3 | 0.3±0.1 | 8±2 | 0.3±0.1 | 12±2 | **0.5±0.1** |
| SM 42:2 | 10±4 | 0.4±0.1 | 16±4 | 0.6±0.2 | 18±6 | **0.7±0.1** |

**Supplementary Table 2: The effect of TGF-β1 and IGF-1 on the level of newly synthesized PL species.** The quantitative values obtained for each stable isotope-labelled PL species were normalized with respect to the cellular protein content and expressed as pmol/mg protein. For each PL species the percentage of stable isotope-labelled PL from the total labelled and unlabelled PL was calculated. Data are presented as means ± SDs (n = 5). Bold data correspond to significantly elevated changes (expressed as fold increases) as shown in Figure 2 and 3. PC = phosphatidylcholine; SM = sphingomyelin.

| PL specie | control | | BMP-2 | | BMP-4 | | BMP-7 | |
|---|---|---|---|---|---|---|---|---|
| | [pmol/mg] | [% labelled] | [pmol/mg] | [% labelled ] | [pmol/mg] | [% labelled ] | [pmol/mg] | [% labelled] |
| PC 30:0 | 31±6 | 3.0±0.6 | 36±7 | 3.6±0.7 | 36±8 | 3.4±0.8 | 40±9 | 3.8±0.8 |
| PC 32:0 | 321±76 | 4.5±1.1 | 343±66 | 5.1±1.0 | 332±91 | 4.7±1.3 | 377±79 | **5.3±0.8** |
| PC 34:0 | 39±10 | 3.9±1.1 | 52±13 | **5.2±0.7** | 47±17 | 4.7±1.9 | 50±12 | 4.9±0.7 |
| PC 32:1 | 116±19 | 4.0±0.8 | 125±18 | 4.5±0.9 | 127±36 | 4.4±1.0 | 141±24 | **4.8±0.7** |
| PC 34:1 | 649±130 | 4.4±1.0 | 726±115 | 5.0±1.2 | 724±168 | 4.9±1.3 | 797±100 | **5.3±1.2** |
| PC 36:1 | 170±41 | 2.7±0.6 | 180±25 | 3.0±0.7 | 183±34 | 3.0±0.9 | 212±20 | **3.4±0.8** |
| PC 34:2 | 261±41 | 6.7±1.4 | 281±32 | 7.4±1.5 | 287±69 | 7.4±1.7 | 315±39 | **7.9±1.2** |
| PC 36:2 | 247±33 | 4.4±1.0 | 274±25 | 4.9±1.3 | 268±40 | 4.8±1.3 | 294±24 | **5.2±1.2** |
| PC 34:3 | 28±6 | 7.9±1.2 | 33±5 | 9.3±1.5 | 32±7 | 9.0±2.0 | 36±7 | 9.7±1.4 |
| PC 36:3 | 161±28 | 5.8±1.3 | 183±31 | 6.7±1.7 | 177±48 | 6.5±1.8 | 197±43 | **7.0±1.5** |
| PC 38:3 | 69±20 | 4.0±1.0 | 74±20 | 4.3±1.1 | 72±20 | 4.2±0.8 | 81±23 | 4.6±0.8 |
| PC 36:4 | 303±107 | 5.4±0.9 | 326±114 | 6.0±0.8 | 335±142 | 5.9±1.2 | 361±130 | **6.3±0.8** |
| PC 38:4 | 390±151 | 4.1±0.8 | 427±169 | **4.6±0.7** | 424±180 | 4.6±0.8 | 462±171 | **4.9±0.6** |
| PC 40:4 | 29±10 | 5.5±1.3 | 32±10 | 6.1±1.3 | 34±14 | 6.3±2.0 | 37±12 | 6.5±1.2 |
| PC 36:5 | 39±11 | 6.3±1.0 | 44±14 | 6.9±1.0 | 44±16 | 7.1±1.3 | 47±14 | **7.2±1.0** |
| PC 38:5 | 238±84 | 5.8±1.2 | 266±96 | 6.5±1.3 | 257±102 | 6.4±1.3 | 281±100 | **6.8±1.2** |
| PC 40:5 | 48±12 | 5.3±0.8 | 52±21 | 5.7±1.6 | 52±19 | 5.8±1.5 | 62±21 | **6.6±1.4** |
| PC 38:6 | 55±17 | 5.5±1.2 | 65±16 | **6.5±1.1** | 58±22 | 6.1±1.5 | 70±22 | **6.6±1.1** |
| PC 40:6 | 33±9 | 4.9±1.0 | 41±14 | 5.9±1.6 | 37±12 | 5.8±1.4 | 45±14 | **6.4±1.3** |
| SM 32:1 | 5±1 | 1.0±0.2 | 5±0 | 1.1±0.1 | 5±1 | 1.1±0.1 | 5±1 | 1.2±0.3 |
| SM 33:1 | 4±0 | 0.7±0.1 | 4±1 | 0.7±0.1 | 4±1 | 0.7±0.2 | 4±1 | 0.7±0.1 |
| SM 34:0 | 3±2 | 0.3±0.2 | 3±2 | 0.3±0.3 | 2±1 | 0.3±0.1 | 3±2 | 0.3±0.2 |
| SM 34:1 | 38±9 | 0.2±0.0 | 41±5 | 0.3±0.0 | 39±11 | 0.3±0.0 | 44±5 | 0.3±0.1 |
| SM 34:2 | 3±1 | 1.0±0.2 | 3±0 | 1.0±0.1 | 4±0 | 1.2±0.2 | 4±0 | 1.2±0.1 |
| SM 35:2 | 7±2 | 0.8±0.2 | 7±1 | 0.9±0.2 | 7±1 | 0.9±0.3 | 6±1 | 0.8±0.2 |
| SM 36:1 | 4±2 | 0.3±0.1 | 5±2 | 0.4±0.1 | 5±2 | 0.3±0.1 | 4±1 | 0.2±0.01 |
| SM 36:2 | 3±1 | 1.7±0.2 | 2±0 | 1.6±0.3 | 3±1 | 1.8±0.2 | 3±0 | 1.6±0.4 |
| SM 42:1 | 5±0 | 0.2±0.1 | 6±1 | 0.2±0.0 | 6±2 | 0.2±0.1 | 8±2 | **0.3±0.1** |
| SM 42:2 | 6±4 | 0.2±0.1 | 6±3 | 0.2±0.1 | 8±3 | 0.3±0.1 | 8±3 | 0.3±0.2 |

**Supplementary Table 3: The effect of BMPs on the level of newly synthesized PL species.** The quantitative values obtained for each of the stable isotope-labelled PL species were normalized with respect to the cellular protein content and expressed as pmol/mg protein. For each PL species the percentage of stable isotope-labelled PL from the total labelled and unlabelled PL was calculated. Data are presented as means ± SDs (n = 5). Bold data correspond to the significantly elevated changes (expressed as fold increases) as shown in Figure 4. PC = phosphatidylcholine; SM = sphingomyelin.
